# Analytical and Clinical Evaluation of a TaqMan Real-Time PCR Assay for the Detection of Chikungunya Virus

**DOI:** 10.1128/spectrum.00088-23

**Published:** 2023-06-05

**Authors:** Anna Andrew, Marimuthu Citartan, Kiing Aik Wong, Thean Hock Tang, Sum Magdline Sia Henry, Ewe Seng Ch'ng

**Affiliations:** a Advanced Medical and Dental Institute, Universiti Sains Malaysia, Kepala Batas, Penang, Malaysia; b Faculty of Medicine and Health Sciences, Universiti Malaysia Sarawak, Kota Samarahan, Sarawak, Malaysia; c Institute of Health and Community Medicine, Universiti Malaysia Sarawak, Kota Samarahan, Sarawak, Malaysia; Quest Diagnostics

**Keywords:** Chikungunya virus, reverse transcription-quantitative real-time PCR, in-house assay, cutoff quantification cycle

## Abstract

Due to the general symptoms presented by the Chikungunya virus (CHIKV)-infected patients, a laboratory test is needed to differentiate CHIKV from other viral infections. The reverse transcription-quantitative real-time PCR (RT-qPCR) is a rapid and sensitive diagnostic tool, and several assays have been developed for detecting and quantifying CHIKV. Since real-time amplification efficiency varies within and between laboratories, an assay must be validated before being used on patient samples. In this study, the diagnostic performance of a TaqMan RT-qPCR assay was evaluated using synthetic RNA and archived patient samples. The cutoff quantification cycle (*C*_q_) value for the assay was determined by experimental evidence. We found the in-house assay was highly sensitive, with a detection limit of 3.95 RNA copies/reaction. The analytical specificity of the assay was 100%. The analytical cutoff *C*_q_ value was 37, corresponding to the mean *C*_q_ value of the detection limit. Using archived samples characterized previously, the sensitivity and specificity of the assay were 76% and 100%, respectively. The in-house assay was also compared with a commercial assay, and we found that the in-house assay had higher sensitivity. Although further evaluation with prospective patient samples is needed in the future, this validated RT-qPCR was sensitive and specific, which shows its potential to detect CHIKV in clinical samples.

**IMPORTANCE** Chikungunya virus causes chikungunya fever, a disease characterized by fever, rash, and joint pain. In the early phase of infection, chikungunya fever is always misdiagnosed as other arbovirus infections, such as dengue. Laboratory tests such as RT-qPCR are therefore necessary to confirm CHIKV infection. We evaluated the performance of an in-house RT-qPCR assay, and our study shows that the assay could detect CHIKV in clinical samples. We also show the cutoff determination of the assay, which provides important guidance to scientists or researchers when implementing a new RT-qPCR assay in a laboratory.

## INTRODUCTION

Chikungunya virus (CHIKV) is an enveloped virus that belongs to the family *Togaviridae* and the genus *Alphavirus*. The CHIKV genome is approximately 12 kb and has two open reading frames (ORFs); the 5′ end has a 7-methylguanosine cap and a polyadenylation signal at the 3′ end. The 5′ ORF is translated from genomic RNA and encodes four nonstructural proteins (nsP1 to nsP4) essential for viral replication and processing. The 3′ ORF is translated from the 26S subgenomic RNA as a single polypeptide, which undergoes cleavage and posttranslational modification to form capsid protein (C), two surface envelope glycoproteins (E1 and E2), and two minor proteins, E3 and 6K (1–3).

Based on the glycoprotein E1 gene phylogenetic analysis, three genotypes of CHIKV were identified, West African, East/Central/South African (ECSA), and Asian (4). A massive chikungunya outbreak caused by the ECSA genotype in the Indian Ocean islands, affecting millions of people, has attracted worldwide attention (5, 6). With globalization and the increase in international travel, CHIKV was introduced to America for the first time in 2013 (7). To date, Asian and ECSA genotypes of CHIKV have been reported in America (8, 9). In Malaysia, a massive outbreak caused by the ECSA genotype occurred in 2008 and 2009, with almost all states reporting CHIKV cases (10).

CHIKV causes chikungunya fever, a disease characterized by fever, rash, and joint pain. Transmitted mainly by Aedes aegypti and Aedes albopictus, the disease has always been misdiagnosed as other viral infections, such as dengue (DENV) and Zika virus (ZIKV), residing in the same vectors. To make matters worse, co-circulation and co-infection of CHIKV, DENV, and ZIKV have been reported (11–14). Differential diagnosis is important because the disease’s severity is different. For instance, DENV infection can lead to dengue shock and hemorrhagic fever, while ZIKV infection has been associated with a newborn malformation (microcephaly) (15, 16). Therefore, a laboratory test is needed to identify the causative agents to help the clinician manage the infection more effectively.

Depending on the duration of the patient's symptoms, the laboratory tests available for CHIKV diagnosis include virus isolation and molecular and serological tests (17). Virus isolation is the gold standard, but this test is seldom done in routine clinical practice. Furthermore, it is time-consuming, as the incubation takes several weeks to a month, and further testing by a molecular or serology test is needed for verification. Most laboratories use serodiagnosis methods to detect immunoglobulin M (IgM) and immunoglobulin G (IgG) antibodies against CHIKV. Although serology tests are simple and straightforward, meta-analysis has shown that their accuracies are relatively low for acute-phase samples (18).

Molecular tests amplifying the target DNA are preferable for the rapid diagnosis of acute CHIKV infection. Conventional polymerase chain reaction (PCR) has been widely used to detect CHIKV (19–22), but it is less sensitive and time-consuming (23). Quantitative real-time PCR (qPCR) is fast and sensitive, and quantifying the virus load can be useful for epidemiological studies. RT-qPCR involves reverse transcription (RT) to synthesize cDNA from the template RNA before amplification to produce multiple copies of double-stranded DNA (dsDNA). As the two-step (reverse transcription and PCR) process is done in one tube, the assay is fast, and the result can be obtained on the same day.

The qPCR systems depend on detecting and quantifying fluorescent signals, which is proportional to the amount of PCR products in a reaction. The fluorescence signal is measured at the end of each amplification cycle, and the quantification cycle (*C*_q_) value represents the cycle at which the fluorescence signal exceeds a defined background threshold. Several TaqMan RT-qPCRs for the detection of CHIKV have been developed (24–30), but the methods used to determine the assay cutoff points (*C*_q_ value) are rarely reported. For instance, Cecilia et al. (31) selected a cutoff *C*_q_ value of 32 based on a nonspecific signal observed in the no-template control, while Edwards et al. (26) followed the cutoff (*C*_q_ value of 37) utilized by another laboratory. Selecting a cutoff value is important to minimize the probability of false-positive or false-negative results (32).

Real-time amplification efficiency varies within and between laboratories. Therefore, the assay should be critically evaluated, and determining the cutoff *C*_q_ value is crucial before its use on clinical samples. In this study, we evaluated the analytical and clinical performance of a TaqMan RT-qPCR for the diagnosis of CHIKV using the primers and probes reported previously (33). An in-house synthetic CHIKV RNA was used to determine the limit of detection (LOD), efficiency, and reproducibility of the assay. The cutoff *C*_q_ value was selected based on experimental evidence. Finally, the clinical performance of the assay was evaluated using archived patient samples that were characterized previously by virus isolation and conventional RT-PCR.

## RESULTS

### Analytical validation and selection of cutoff.

**FIG 1:**
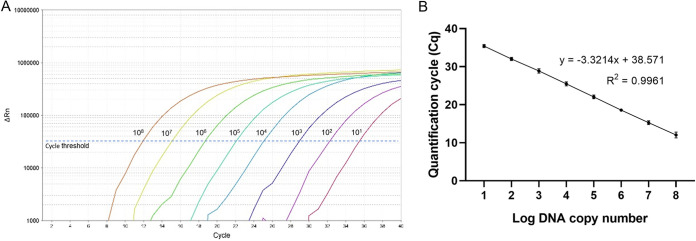
Amplification profile (10^8^ to 10^1^ RNA copies) (A) and calibration curve (B) for the CHIKV TaqMan RT-qPCR generated from the *C*_q_ values obtained against the known concentration of 10-fold serially diluted synthetic RNA ranging from 10^8^ to 10^1^ RNA copies/reaction (logarithm values of the absolute amount of RNA copy number).

The amplification results from synthetic CHIKV RNA ranged from 1 × 10^8^ to 1 × 10^1^ copies/reaction (Fig. 1A), and the efficiency (*E*) of the RT-qPCR was 100%. The calibration curve was linear (Fig. 1B), with a correlation coefficient (*R*^2^) of 0.996, and the slope was −3.3214. The LOD of the assay was 3.95 RNA copies/reaction, which was the last dilution to be amplified with a detection rate of 100% (Table 1). The analytical cutoff *C*_q_ value was 37, corresponding to the mean *C*_q_ value of the LOD (Table 1). For the interpretation of a sample, the *C*_q_ value of ≤37 is interpreted as positive and >37 as negative.

**TABLE 1:** Mean *C*_q_ value and the total number of positive replicas for each RNA dilution used in the determination of LOD

RNA copies/reaction	*C*_q_ value^*a*^	No. of positive replicas/total no. of replicas
1.58 × 10^1^	35.42 ± 0.40	7/7
7.9	36.53 ± 0.37	7/7
3.95	37.20 ± 0.46	7/7
1.975	37.62 ± 0.44	4/7

a Data are shown as mean ± standard deviation (SD).

The coefficient of variation (CV) of the intra-assay and inter-assay ranged between 0.95 to 3.37% and 0.25 to 4.28%, respectively (Table 2). All the dengue-positive and healthy human serum tested negative, which showed 100% analytical specificity. BLAST analysis showed that the primers did not match the sequence of other alphaviruses, such as the O'nyong Nyong (ONN) virus, Semliki Forest virus (SFV), and Ross River virus (RRV).

**TABLE 2:** Intra- and inter-assay reproducibility of the CHIKV TaqMan RT-qPCR^*a*^

No. of RNA copies/reaction	Intra-assay variation^*b*^		Inter-assay variation^*c*^	
	*C*_q_ ^*d*^	CV (%)	*C*_q_ ^*d*^	CV (%)
1.58 × 10^8^	11.62 ± 0.39	3.37	12.12 ± 0.52	4.28
1.58 × 10^7^	15.28 ± 0.52	3.37	15.28 ± 0.10	0.68
1.58 × 10^6^	18.78 ± 0.25	1.33	18.56 ± 0.34	1.87
1.58 × 10^5^	22.38 ± 0.43	1.91	22.01 ± 0.40	1.83
1.58 × 10^4^	25.59 ± 0.33	1.28	25.46 ± 0.18	0.69
1.58 × 10^3^	28.84 ± 0.70	2.43	28.88 ± 0.07	0.25
1.58 × 10^2^	32.15 ± 0.42	1.31	32.01 ± 0.35	1.09
1.58 × 10^1^	35.65 ± 0.34	0.95	35.36 ± 0.33	0.92

a CV, coefficient of variation; *C*_q_, quantification cycle.

b Intra-assay variation was determined on three replicates in the same PCR run.

c Interassay variation was calculated on values obtained in three separate runs.

d Mean ± standard deviation (SD).

### Diagnostic performance of the in-house RT-qPCR.

From a total of 37 CHIKV-positive samples, 76% (*n* = 28) were tested positive by the in-house RT-qPCR. None of the CHIKV-negative samples was positive by the in-house assay, which shows 100% specificity (Table 3).

**TABLE 3:** Diagnostic performance of the TaqMan RT-qPCR^*a*^

Index test result	Reference standard (RT-PCR and/or virus isolation)		Total
	No. positive	No. negative	
In-house RT-qPCR			
No. positive	28	0	28
No. negative	9	11	20
Total	37	11	48

a Sensitivity was 76% (95% confidence interval [CI], 59 to 88), and specificity was 100% (95% CI, 71 to 100).

### Comparison of the in-house RT-qPCR with a commercial RT-qPCR.

The diagnostic performance of the in-house RT-qPCR was compared to a commercial RT-qPCR (Table 4). The sensitivity of the in-house RT-qPCR (79%) was shown to be higher than the commercial assay (41%). Both assays showed 100% specificity, with no cross-reactivity with the dengue-positive samples.

**TABLE 4:** Diagnostic performance comparison of the in-house and commercial RT-qPCR assays

Test type	Reference test (RT-PCR and/or virus isolation)	
	Positive	Negative
In-house RT-qPCR^*a*^		
No. positive	23	0
No. negative	6	4
Genesig RT-qPCR^*b*^		
No. positive	12	0
No. negative	17	4

a Sensitivity was 79% (95% CI, 60 to 92), and specificity was 100% (95% CI, 40 to 100).

b Sensitivity was 41% (95% CI, 24–61), and specificity was 100% (95% CI, 40 to 100).

The percentage of agreement between the in-house and commercial RT-qPCR was evaluated. Based on Table 5, the positive percent agreement (PPA) and negative percent agreement (NPA) were 100% and 47.6%, respectively. The overall rates of agreement were 66.7%.

**TABLE 5:** Percentage agreement between in-house RT-qPCR and the commercial RT-qPCR assays^*a*^

Index test result	Commercial RT-qPCR	
	No. positive	No. negative
In-house RT-qPCR		
No. positive	12	11
No. negative	0	10
Total	12	21

a Positive percentage agreement was calculated as (12/12) × 100% = 100%, negative percentage agreement was calculated as (10/21) × 100% = 47.6%, and overall percentage agreement was calculated as (22/33) × 100% = 66.7%.

## DISCUSSION

The unprecedented outbreaks in the Indian Ocean outbreak (6) and India (34) have caught the attention of researchers from all over the world. One of the contributing factors to the high magnitude of the disease is the delay in diagnosis and reporting (35). Since then, there has been a significant increase in the development of CHIKV diagnostic tests to correctly diagnose the disease (36). One of the tests is the RT-qPCR, a rapid, sensitive, and specific assay that can be easily integrated into a modern lab.

In-house or commercial RT-qPCR assays for the detection of CHIKV are widely available in the literature. Although these tests have been optimized, the different parameters used in laboratories, such as reagents or real-time machines, might affect the assay performance (37). Therefore, a diagnostic assay used for the first time in a laboratory should be evaluated carefully to ensure it can accurately diagnose disease. In this study, we evaluated a TaqMan RT-qPCR developed by Wang et al. (33) prior to use on patient samples. We used in-house-prepared synthetic RNA to evaluate the analytical performance of the assay since standard reference RNA was unavailable. Our result showed that the assay was reproducible with high accuracy and precision (*E* = 100%, *R*^2^ > 0.99). The analytical evaluation can exclude factors such as the presence of inhibitors, reagent discrepancy, or pipetting errors that can affect the efficiency of the assay (38).

As reviewed by Caraguel et al. (39), the current World Organization of Animal Health (OIE) guideline defines analytical sensitivity as the endpoint dilution at which 50% of the tested samples are positive. In their review, for a 99% level of confidence, at least 1 negative result out of the 7 replicates for a serial dilution concentration indicates the likelihood of testing positive as lower than 50% (39). Therefore, LOD was determined as the lowest dilution at which the assay detected positive signals on all 7 replicates. The LOD of the in-house was 3.95 RNA copies/reaction, similar to the previous LOD (33). The same group of researchers used the same primers and tested on SYBR green I-based real-time RT-PCR assay to detect CHIKV (40). In concordance, a similar LOD (4.12 RNA copies) was reported. Their study used the CHIKV Asian genotype as the positive control, while our study used the ECSA genotype. These two studies suggest that the primer pairs could detect two CHIKV genotypes with similar sensitivity.

The interpretation of the qPCR assay depends on the fluorescence emitted during the PCR cycle, which must cross the threshold, which is the *C*_q_ value or the cycle number. In most reports, the selection of the cutoff value for qPCR was subjective and without evidence (26, 31). The cutoff *C*_q_ value can be different between laboratories since it is very difficult to use a standardized protocol. It is crucial to determine the cutoff *C*_q_ value since different sampling and extraction protocols, amplification conditions, and detection methods may compromise the efficiency of the assay. We used the experimental approach, whereby the analytical cutoff selected in this study was based on the mean *C*_q_ value of the defined LOD.

Analytical specificity is the ability of an assay to distinguish a target analyte despite the presence of other components in the sample (38). None of the dengue-positive samples was detected using the in-house RT-qPCR, which showed that the assay was highly specific to CHIKV. Furthermore, BLAST analysis confirmed no sequence similarities between the primers and the genome sequence of the three related alphaviruses (ONN, SFV, and RRV).

The diagnostic accuracy of the in-house RT-qPCR was evaluated using archived patient samples collected during an outbreak in Sarawak in 2009. Archived samples were used because there was no recent outbreak in Malaysia, and it is difficult to access the sporadic cases samples. However, these samples were collected in a batch, and thus, it has better control over the population variation that may affect the assay's outcome. The sensitivity and specificity of the assay were 76% and 100%, respectively. The assay sensitivity evaluated in this study was slightly lower than that of other reported TaqMan RT-qPCRs (98% to 100%) (26, 41). The moderate sensitivity of the assay is expected because these archived samples were kept at −20°C for more than 10 years. The very long storage duration could have resulted in RNA degradation. Unlike DNA, RNA is less stable and susceptible to hydrolysis, especially at high temperatures (42). The phenomena of RNA degradation were also supported by the low RNA concentration detected in this batch of samples (ranging from 3.1 × 10^3^ to 1.2 × 10^1^ RNA copies), which is lower than the RNA concentration reported previously. The RNA concentration in the acute phase samples is usually between 10^3^ to 10^8^ RNA copies/mL (28, 43–45). Despite the RNA degradation issue, our assay showed high specificity and no cross-reactivity with dengue-positive samples.

We compared the diagnostic performance of the in-house assay with a commercial assay available in our lab. With primary tests (RT-PCR and virus isolation) as the reference standard, the in-house assay had a higher sensitivity than the commercial assay. The agreement between the two assays was investigated, and we found that the PPA was 100% and the NPA was 47.6%. The low NPA was due to the 11 samples that tested negative by commercial assay but positive by in-house TaqMan RT-qPCR. These 11 samples were positive by the primary tests (i.e., virus isolation and RT-PCR), which shows that the commercial assay has low sensitivity. The low sensitivity can be explained by the low RNA concentration of this batch of samples, as mentioned previously. Another possible cause of the commercial assay's low sensitivity is the mutation-prone property of the CHIKV genome, which could have mutated over the years that resulted in the poor binding of the mismatched primers. However, the mismatches of the primers supplied in the commercial test to the different CHIKV genotypes were not investigated since the manufacturer did not reveal the primer sequence. As such, the commercial assays must be frequently tested to ascertain the binding capacity of the primers. Another way is to design two or more sets of primers. For instance, one study developed RT-qPCR using two sets of primers and probes to maximize the detection of different CHIKV strains (24). Nevertheless, similar to the TaqMan RT-qPCR evaluated in this study, the commercial assay is highly specific, and no cross-reactivity with dengue-positive samples was recorded.

Since it is not feasible to get CHIKV patient samples, we use archived patient samples stored at −20°C. These samples can introduce bias (46), and deterioration of the samples can increase the risk of false results. In addition, the small sample size used to evaluate the diagnostic accuracy of a test can produce an imprecise estimate (the wide 95% confidence interval shown in Table 3). Therefore, to accurately evaluate the diagnostic performance of an assay, it is recommended to use prospective patient samples with sufficient sample size. The sample size can be estimated using a simple nomogram (47) or software (48). The assay's specificity can be further verified with other arboviruses, such as the Zika virus and yellow fever virus, in which the disease is usually presented with similar clinical presentation, especially at the early stage of infection in the region whereby co-circulation and co-infection of these viruses are prevalent.

### Conclusion.

In summary, we evaluated the analytical and clinical performance of an in-house RT-qPCR assay. With in-house prepared synthetic RNA, we have shown that the assay has superior analytical sensitivity and specificity. We also demonstrated that the cutoff *C*_q_ value selection was based on experimental evidence following the latest guideline by OIE. The moderate sensitivity of the assay was expected due to the long storage of archived samples at −20°C. Nevertheless, the in-house assay had better diagnostic performance than a commercial assay. This in-house TaqMan RT-qPCR can detect low viral load (3.95 RNA copies/reaction), which makes it a sensitive tool for detecting and quantifying CHIKV in patient samples. The quantification of CHIKV load is a useful indicator of active infection.

## MATERIALS AND METHODS

### CHIKV culture.

CHIKV was isolated from a patient sample collected during the 2009 CHIKV outbreak in Sarawak. Briefly, 10 μL of the patient serum was inoculated into the Vero cell line (Dulbecco’s modified Eagle medium [DMEM] supplemented with 5% final fetal calf serum). The culture supernatant was harvested when a complete cytopathic effect was seen. Next, a second passage was prepared on a large scale to keep the virus stock. The viral inocula used in this study were prepared in the third passage. The partial E1 gene of the virus genome was verified by DNA sequencing.

### RNA extraction.

Viral RNA was extracted from 200 μL of CHIKV culture supernatant using the High Pure viral RNA extraction kit (Roche Diagnostics Systems, Branchburg, NJ, USA) according to the manufacturer's protocol. The RNA was eluted in 50 μL of sterile ultrapure water and was stored at −80°C for further use.

### Preparation of synthetic CHIKV RNA.

A standard RT-PCR was carried out using the CHIKV T7 forward primer (5′-TAATACGACTCAC TATAGGGCTCATA CCGCATCCGCATCAG-3′) and CHIKV reverse primer (5′-ACATTGGCC CCACAATGAATTTG-3′). *In vitro* transcription (IVT) of the amplified PCR product (1 μg) was done using TranscriptAid T7 high-yield transcription kit (Thermo Scientific, MA, USA). Briefly, the DNA products were subjected to IVT for 2 h at 37°C. The IVT products were treated with 1 U of DNase I supplied in the Turbo DNase kit (Thermo Scientific, MA, USA) by incubation at 37°C for 15 min to remove the remaining DNA. The DNase activity was stopped by incubation with DNase inactivation reagent supplied in Turbo DNA-free kit (Thermo Scientific, MA, USA) for 5 min at room temperature. Then, the resulting IVT products were purified with a QIAquick PCR purification kit (Qiagen, Heiden, Germany). The concentration of the RNA was measured using the Qubit RNA broad-range (BR) assay kit (Thermo Scientific, MA, USA) according to the manufacturer's protocol. The RNA transcripts copy number was calculated using the following equation:

$$\text{RNA copies}/\mu \text{L }=\frac{\text{6}.0\text{22 }\times \text{ 1}0^{\text{23}}\;\left(\text{copies}/\text{mol}\right)\;\times \text{ concentration }\left(\text{g}/\mu \text{L}\right)}{\text{MW }\left(g/\text{mol}\right)}$$

where MW is the molecular weight of single-stranded RNA and is calculated as 129 (length of RNA transcript in nucleotide) × 340 Da/base (approximate weight of a single-stranded nucleotide). The synthetic RNA was stored at −80°C for future use.

### TaqMan RT-qPCR.

The primers and a fluorescent-labeled TaqMan probe (5′FAM-3′BHQ) that target the glycoprotein E1 genome region were used in this assay (33). The one-step TaqMan RT-qPCR was carried out according to the method described by Wang et al. (33) with slight modifications. GoTaq probe 1-step RT-qPCR system (Promega, Lyon, France) was used and performed in a final volume of 20 μL containing 5 μL of extracted RNA, 10 μL of 2× GoTaq probe qPCR master mix with dUTP, 200 nM of the TaqMan probe, 200 nM of each primer, and 0.4 μL of GoScript RT mix for 1-step RT-qPCR. Real-time RT-PCR assays were carried out using QuantStudio 5 real-time PCR system (Applied Biosystems, Foster City, CA, USA) with the following steps: reverse transcription at 50°C for 30 min, DNA polymerase activation at 95°C for 15 min 40 cycles of PCR at 95°C of denaturation for 30 s, and 58.7°C of annealing/extension for 1 min. The fluorescence was measured during the annealing/extension step. In each run, synthetic RNA was included as a positive control for monitoring assay variation, and a no-template control (NTC) served as a negative control. The run is invalid when there is an amplification of NTC. The data were analyzed by QuantStudio Design and Analysis v1.5.2 software, and the calibration curve was generated with GraphPad Prism v6.05 software (GraphPad Software Inc., CA, USA).

### Analytical validation and selection of cutoff *C*_q_ value.

The linearity of the assay was determined by using synthetic RNA diluted at a 10-fold serial dilution in CHIKV-negative serum (10^8^ to 10^1^ copies/reaction). A total of three reactions were conducted per dilution. A calibration curve was established by plotting the mean *C*_q_ values (*y* axis) against the log concentration of synthetic CHIKV RNA (*x* axis). The PCR efficiency (*E*) was measured with the formula 10^(−1/slope)^. The intra-assay variation was calculated based on the triplicate *C*_q_ values of each dilution. The same operator repeated the experiment three times over consecutive days, using the same batch of reagents to determine inter-assay variation.

Further dilution (2-fold serial dilution) of the lowest RNA concentration from the linear range study was prepared to refine the estimation of the detection limit. Seven replicates were prepared for each dilution, and the LOD is the lowest dilution at which the assay detected a positive signal on all the replicates. Meanwhile, the analytical cutoff point was selected based on the mean *C*_q_ value corresponding to the LOD (39).

The analytical specificity of the assay was evaluated with 10 dengue-positive samples and 10 healthy human samples. These samples were previously characterized and confirmed by Lim et al. (49). The analytical specificity was also verified by nucleotide BLAST of the primer and probe sequence against closely related alphaviruses such as O’nyong Nyong virus, Semliki Forest virus, and Ross River virus.

### Diagnostic performance of the in-house RT-qPCR.

A total of 48 clinical serum samples were subjected to the diagnostic performance of the TaqMan RT-qPCR. These samples were collected during the CHIKV outbreak between June and December 2009 and were sent to the Institute of Health and Community Medicine, University Malaysia Sarawak, for laboratory investigation. Conventional RT-PCR and virus isolation were used to characterize these archived samples. The patient samples were kept at −20°C due to the insufficient storage in the −80°C freezer available during this period. Ethical approval was obtained from the Medical Research Ethics Committee, Universiti Malaysia Sarawak (ethics reference number FME/22/69).

In this study, 48 samples were chosen randomly based on the available volume (minimum, 200 μL) in each sample. We defined a sample as positive when one or both tests (conventional RT-PCR and virus isolation) were positive and a sample as negative when both tests were negative. Based on the primary data, 37 positive and 11 negative samples were chosen. Viral RNA was extracted from 200 μL of each sample, and RT-qPCR was carried out using the same protocol mentioned above.

### Assay comparison with a commercial real-time RT-PCR.

The performance of the TaqMan RT-qPCR assay was compared with a commercial RT-qPCR (Genesig standard kit; Primerdesign, UK). Due to the limited volume of some samples, only a subset of the patient samples was evaluated. Of the 33 samples, 29 were positive and 4 were negative by primary tests (RT-PCR and virus isolation). Ten dengue-positive samples were also evaluated. The amplification was carried out as suggested by the manufacturer with slight modifications by using the GoTaq Probe 1-step RT-qPCR system (Promega, Lyon, France). Five microliters of the extracted RNA, 10 μL of 2 × GoTaq probe qPCR master mix with dUTP, 1 μL of CHIKV primer/probe mix, 1 μL of GoScript RT mix, and 3 μL of RNase/DNase-free water were added to a final reaction volume of 20 μL. The RT-PCR was performed using QuantStudio 5 real-time PCR system. The PCR conditions were set following the manufacturer's instructions. The positive control supplied in the kit and NTC were included in each run. In-house and commercial RT-qPCR assays were run using the same batch of the extracted RNA on the same day to rule out discrepancies due to the quality of the samples. The diagnostic performance of the commercial assay was compared with in-house RT-qPCR. In addition, the positive and negative percent agreement (PPA and NPA, respectively) and the overall rate of agreement of the two assays were determined as described previously (50).

### Data availability.

The sequence of the partial E1 gene of the virus genome is available in GenBank (accession number OP265748).
